# Could *let-7f*, *miR-10b*, *miR-34a*, *miR-181b*, and *miR-181d* Be Useful Tools as a Target Therapy for Uterine Leiomyosarcoma?

**DOI:** 10.3390/biomedicines13030560

**Published:** 2025-02-23

**Authors:** Bruna Cristine de Almeida, Laura Gonzalez dos Anjos, Luciane Tsukamoto Kagohara, Ayman Al-Hendy, Qiwei Yang, Edmund Chada Baracat, Cláudia Malheiros Coutinho-Camillo, Katia Candido Carvalho

**Affiliations:** 1 Laboratório de Ginecologia Estrutural e Molecular (LIM 58), Disciplina de Ginecologia, Departamento de Obstetricia e Ginecologia, Hospital das Clinicas da Faculdade de Medicina da Universidade de Sao Paulo (HCFMUSP), Cerqueira Cesar, São Paulo 05403-010, Brazil; bruc_10@hotmail.com (B.C.d.A.); lauragonzalezanjos@gmail.com (L.G.d.A.); edmund.baracat@hc.fm.usp.br (E.C.B.); 2 Sidney Kimmel Comprehensive Cancer Center, Johns Hopkins University, Baltimore, MD 21231, USA; ltsukam1@jhmi.edu; 3 Department of Obstetrics and Gynecology, University of Chicago, Chicago, IL 60637, USA; aalhendy@bsd.uchicago.edu (A.A.-H.); yangq@bsd.uchicago.edu (Q.Y.); 4 International Research Center, A.C.Camargo Cancer Center, Rua Taguá 440, São Paulo 01508-010, Brazil; ccamillo@accamargo.org.br

**Keywords:** uterine leiomyosarcoma, uterine leiomyoma, microRNAs, uterine mesenchymal tumors, gene expression, epigenetics, *miR-34a*, *miR-181b*

## Abstract

**Background/Objectives:** We have previously identified *let-7f-5p*, *miR-10b-5p*, *miR-34a-5p*, *miR-181b-5p*, and *miR-181d-5p* as differentially expressed between uterine leiomyoma (LM) and leiomyosarcoma (LMS) tissue samples. The present study aimed to characterize these miRNA expression profiles and to assess the functional role of *miR-34a* and *miR-181b* in uterine LM and LMS cells. **Methods:** All the selected miRNAs showed downregulation in LMS cells compared to LM cells, but only *miR-34a* and *miR-181b* expression patterns matched those of patient samples. Therefore, these two miRs were selected for further analyses. **Results:** Loss of function analysis demonstrated that *miR-34a* and *miR-181b* silencing inhibited LM cell proliferation and migration. *MiR-34a* silencing induced *CCND1* and *MDM4* expression and inhibited *KMT2D*, *BCL2*, and *NOTCH2* in LM. Silencing of *miR-181b* promotes *TIMP3* and *FGFR1* expression in LM and diminishes *BCL2*, *NOTCH2*, *ATM*, *IRS1*, and *PRLR*. Gain of function analysis revealed that the introduction of *miR-34a* and *miR-181b* mimics suppressed proliferation and migration in malignant LMS cells. Additionally, transfection with a *miR-34a* mimic downregulated *NOTCH2* and *BCL2* expression and enhanced the expression of *CCND1*, *KMT2D*, and *TP53* in LMS cells. Moreover, *miR-181b* overexpression decreased *TIMP3*, *NOTCH2*, *ATM*, and *IRS1* expression and increased the expression of *FGFR1* in this cell. Importantly, the single introduction of either a *miR-34a* or *miR-181b* mimic was able to decrease the invasion capacity of LMS cells. **Conclusions:** Our studies demonstrated that *miR-34a* or *miR-181b* may play an anti-oncogenic role in uterine tumors; further studies are needed to better understand the role and regulatory mechanism of these miRNAs in LMS cancer development, which will help provide prognostic and therapeutic options for patients with LMS.

## 1. Introduction

Benign and malignant neoplasms from the uterine body are classified according to their morphologic features (cell physiognomies, stromal components, tumor architecture, and growth patterns) [1]. Nevertheless, uterine mesenchymal tumors (UMTs) may present morphologic and immunohistochemical overlapping [2]. Leiomyoma (LM) is a very common benign variant of UMTs that affects almost 80% of women of reproductive age, and its highest incidence occurs in Afro-descendant women [3,4,5,6,7]. Leiomyosarcoma (LMS) is a heterogeneous malignant variant that accounts for 1–2% of all uterine malignancies and over 50% of all uterine sarcomas [3,8,9]. LMS cell composition is characterized by high cellularity with frequent nuclear atypia and a mitotic index, enrichment for malignant smooth muscle cells, invasion, and necrosis. It is associated with a worse prognosis due to its frequent recurrences (53–75%) and metastasis [8,10,11]. Unlike LM, LMS develops independently of hormonal status or oral hormonal contraceptive use [12] and may arise due to enhancing the chromosomal instability and overexpression of genes linked to cell proliferation [13,14].

The differential diagnosis between LM and uterine LMS is still challenging for pathologists [2] and often cannot be performed using clinical criteria only [2]. Some subtypes of LM present similar features to LMS, such as severe cytological atypia, increased mitotic activity, high cellularity, and necrotic areas [1,15,16,17,18]. Unconventional LM cannot be clinically distinguished from LMS by imaging techniques [4,19,20]. These facts motivate the constant search for new biomarkers that would facilitate the discrimination between these tumors and avoid misdiagnosis, which will significantly reduce treatment delays and morbidity [2,8].

Among all molecular alterations found in LMS so far, epigenetic processes may strongly influence cellular phenotype changes by dysregulating gene expression [21,22]. Epigenetic alterations are considered major regulation mechanisms during tumor progression and tumorigenesis [10,21,22]. MiRNAs act as post-transcriptional regulators of protein-encoding genes [10,21,23] by binding the 3′ UTR of their target genes [24,25]. Through those processes, they can control numerous biological activities. A specific miRNA can play both tumor suppressor and oncogenic roles according to its function in a specific tissue [8,24,25]. In this sense, the well-known tumor suppressor *miR-34a* negatively regulates several processes associated with tumorigenesis; however, when upregulated, it may act as a tumor promoter in some tissues [26]. In some circumstances, *miR-34a* inhibits cell proliferation or induces a senescence phenotype and apoptosis [27]. This scenario was also identified with the pro-inflammatory miRNA *miR-181b*, the functional significance of which depends on the tumoral context [28].

Expression and processing of miRNAs are necessary for differentiating mesenchymal lineages, including osteocytes, adipocytes, skeletal muscle, and cardiac muscle [29]. Both LM and LMS present epigenetic alterations associated with miRNAs directly related to their development [4,19,20,30,31,32,33,34]. Our group has examined the role of miRNAs as potential molecular biomarkers for differentiating LM from LMS and better understanding the differential diagnosis, prognosis prediction, and treatment options [4,19,20]. Characterization of miRNAs with differential expression in UMTs has been an important focus of our studies. Additionally, we previously validated the expression profile of hundreds of miRNAs potentially related to cancer initiation in different types of UMTs [8,10,35,36]. However, their function in LMS development is unknown. In this study, we selected five validated miRNAs (*let-7f*, *miR-10b*, *miR34a*, *miR-181b*, and *miR-181d*) with differential expression patterns between LM and LMS tissues and compared their expression patterns between LMS and LM cells. Furthermore, we employed functional analysis to determine their role and regulated targets in LM and LMS cells.

## 2. Materials and Methods

### 2.1. Cell Culture and miRNA Isolation

PCS-460-011 (myometrium), THESCs–CRL-4003 (LM), and SK-UT-1–HTB-114 (LMS) cells obtained from the American Type Culture Collection (ATCC, Manassas, VA, USA) were grown in specific mediums and conditions as previously described [8]. Cell authentication was performed by DNA short tandem repeat analysis using the GenePrint 10 System (Promega, Madison, WI, USA), following the manufacturer’s recommendations. Gene Mapper V. 4.1 software was used for data analysis that was based on similarities and was checked against the database provided by the ATCC Search the STR Database [8,37]. In addition, the cells were tested for mycoplasma [38].

RNA extraction from cell lines was performed using the mirVana miRNA Isolation extraction kit (Ambion, Foster City, CA, USA) according to the manufacturer’s instructions. RNA and miRNA concentrations and purity were verified by a NanoDrop 2000 spectrophotometer (Thermo Fisher Scientific, Carlsbad, CA, USA) and a Qubit 2.0 fluorometer (Thermo Fisher Scientific), as previously described.

### 2.2. Complementary DNA (cDNA) Synthesis and Quantitative PCR (qPCR) for miRNA Detection

The set of miRNAs in the present study was selected based on our results from previous studies with miScript miRNA PCR Arrays, MIHS-102Z-Qiagen, and the MIHS-109ZA-Qiagen 96-well plate panel (Qiagen, Hilden, Germany) [8,10,35,36]. The TaqMan Advanced miRNA cDNA Synthesis kit (Applied Biosystems, Waltham, MA, USA) was used for cDNA synthesis. The expression profile of miRNAs was assessed by qPCR. Reactions were performed with the TaqMan Fast Advanced Master Mix and TaqMan Advanced miRNA Assay (Applied Biosystems, Waltham, MA, USA), following the manufacturer’s instructions. The specific TaqMan assays used in the reactions for selected miRNAs were *let-7f-5p* (478578_mir), *miR-10b-5p* (478494_mir), *miR-34a-5p* (478048_mir), *miR-181b-5p* (478583_mir), and *miR-181d-5p* (479517_mir). In addition, *miR-320a* (478594_mir) was used as the best normalizer, as predicted by the RefFinder algorithm analysis software version 1 [39].

The qPCR amplifications were performed in an ABI 7500 Real-Time PCR instrument (Thermo Fisher Scientific). Quantification of transcripts was performed using the 2^−∆∆Ct^ method and SDS 3.0 software (Applied Biosystems, Waltham, CA, USA). MiRNA expression values are described as fold change (FC) or fold regulation (FR). To identify differentially expressed miRNAs, a cut-off value for miRNA expression analysis was set at >1 for FC and at ≥+1 and ≤1 for FR.

### 2.3. Cell Transfection with Mimic and Small Interfering RNA (siRNA)

*MiR-34a* and *miR-181b* sequences for expression induction (HMI0508 and HMI0270 MISSION microRNA mimic, Sigma Aldrich, St. Louis, MO, USA) and inhibition (HSTUD0508 and HSTUD0270 MISSION Synthetic miRNA Inhibitors, siRNA—Sigma Aldrich, MO, USA), as well as a MISSION esiRNA EGFP used as a control (EHUEGFP, Sigma Aldrich, MO, EUA), were synthesized. THESCs and SK-UT-1 cells (2 × 10^5^ cells/well) were seeded in a 24-well plate and transfected with Lipofectamine RNAiMAX Transfection Reagent (Invitrogen, Carlsbad, CA, USA) in a reduced-medium Opti-MEM I Reduced Serum Medium (Gibco, Carlsbad, CA, USA) without antibiotics, according to the manufacturer’s instructions. Mimic siRNAs for *miR-34a* and *miR-181b*, as well as EGFP siRNA, were diluted to 10 µM and used at a final concentration of 1 pmol for 96-well plates, 5 pmol for 24-well plates, and 25 pmol for 6-well plates for both cells. The efficiency of mimic and silencing siRNAs was evaluated at 24, 48, 72, and 96 h after transfection by qPCR using FC. The assay was carried out in quadruplicate and in two independent experiments.

### 2.4. Proliferation Assay

THESCs and SK-UT-1 cells were seeded in 96-well plates (2 × 10^4^/well) and transfected with mimic siRNA for *miR-34a* and *miR-181b* and EGFP siRNA (negative control) as previously described. After transfection at certain time points, cell viability was measured using PrestoBlue Cell Viability Reagent (Invitrogen, CA, USA) according to the manufacturer’s instructions. The intensity of the fluorescence was measured using the GloMax-Multi Detection System (Promega, Madison, WI, USA) [40]. Two independent experiments were performed in quadruplicate.

### 2.5. Scratch Assays

THESCs (2 × 10^5^/well) and SK-UT-1 (3 × 10^5^/well) cells were seeded in 24-well plates. A plastic p200 pipette tip (Fisher) was used to scratch the cell monolayer to create a cleared area before transfections [40,41]. Cells were transfected with mimic siRNA for *miR-34a* and *miR-181b*, as well as EGFP siRNA, as previously described and refed with fresh growth media containing 1% FBS. Microphotographs were taken at the established times with an inverted optical Zeiss microscope (AxioCam ERc 5c, Wetzlar, HE, Germany) and analyzed by ImageJ software (V1.53b, Bethesda, MD, USA) [40]. This assay was performed in two independent experiments in quadruplicate.

### 2.6. Invasion Assays

To evaluate the invasion ability of SK-UT-1 cells, 2 × 10^6^ cells/well in six-well plates were seeded and transfected with mimic siRNA for *miR-34a* and *miR-181b*, as well as EGFP siRNA, as previously described. Cells were incubated for 2 h with Basal Medium Eagle (Sigma-Aldrich, Darmstadt, Germany) medium supplemented with 1% FBS as described by Laurentino et al. [42]. After the incubation, 2 × 10^6^ cells per well were seeded in transwell inserts with a pore size of 8 µm (BD Biosciences, Becton Dickinson) containing Geltrex (Thermo Fisher Scientific) rehydrated with medium containing 1% FBS. Medium containing 10% FBS was used as a chemoattractant, and cells were incubated for 24 h. Then, 4% paraformaldehyde was used to fix the invading cells that were stained with 0.2% crystal violet [42]. Microphotographs of inserts were taken in an inverted optical Nikon Eclipse TE200 microscope (Nikon Instruments Inc., Melville, NY, USA), and the invading cells were quantified and analyzed using ImageJ software (V1.53b, Bethesda, MD, USA). Two independent experiments were performed in quadruplicate.

### 2.7. In Silico Analysis

Networks of genetic interactions of *miR-34a* and *miR-181b* were evaluated by in silico (computational) analyses on the TargetScanHuman 7.0 [43], miRTargetLink 2.0 [44], and miRDB–MicroRNA Target Prediction Database [45,46,47] databases, respectively. These analyses allowed the prediction of their main target genes, as well as predictors that are strongly related to the tumorigenic process. In this analysis, the common target genes among the data banks were considered, and the relevant targets with available assays in our laboratory were selected for qPCR assays.

### 2.8. Complementary DNA (cDNA) Synthesis and qPCR for Target Gene Detection

RNA extraction was performed as previously described, and cDNA synthesis was carried out using the High-Capacity cDNA Reverse Transcription kit (Thermo Fisher Scientific, Vilnius, Lithuania) according to the manufacturer’s instructions. Gene expression assays were performed with the TaqMan Universal PCR Master Mix and TaqMan Gene Expression assay (Applied Biosystems, CA, USA), which inventoried probes for each gene, as recommended by the manufacturer. Additional details are given in the Supplementary Materials (Table S1). Human *B2M* (4326319E) was used as a housekeeping gene [39]. This assay was performed in two independent experiments in duplicate.

### 2.9. Statistical Analysis

Initially, the Shapiro–Wilk normality test was used to verify the assumption of normality of numerical information. A student’s *t*-test or analyses of variance (ANOVA) were used to compare groups for parametric variables and the Mann–Whitney U or Kruskal–Wallis tests were used for non-parametric variables. MiRNA expression levels between two groups (LM and LMS) were evaluated with the Mann–Whitney U non-parametric test. For multiple comparisons, Kruskal–Wallis’s test with Dunn’s multiple comparison post-test was applied. An alpha significance level of 5% was used for the inferential analyses. Data were entered in Excel 2013 for Windows, and statistical analyses were performed using GraphPad Prism 5.0 (GraphPad Software, San Diego, CA, USA) and SPSS version 21 (IBM Corp., Armonk, NY, USA) for Windows [10].

## 3. Results

**Figure 1:**
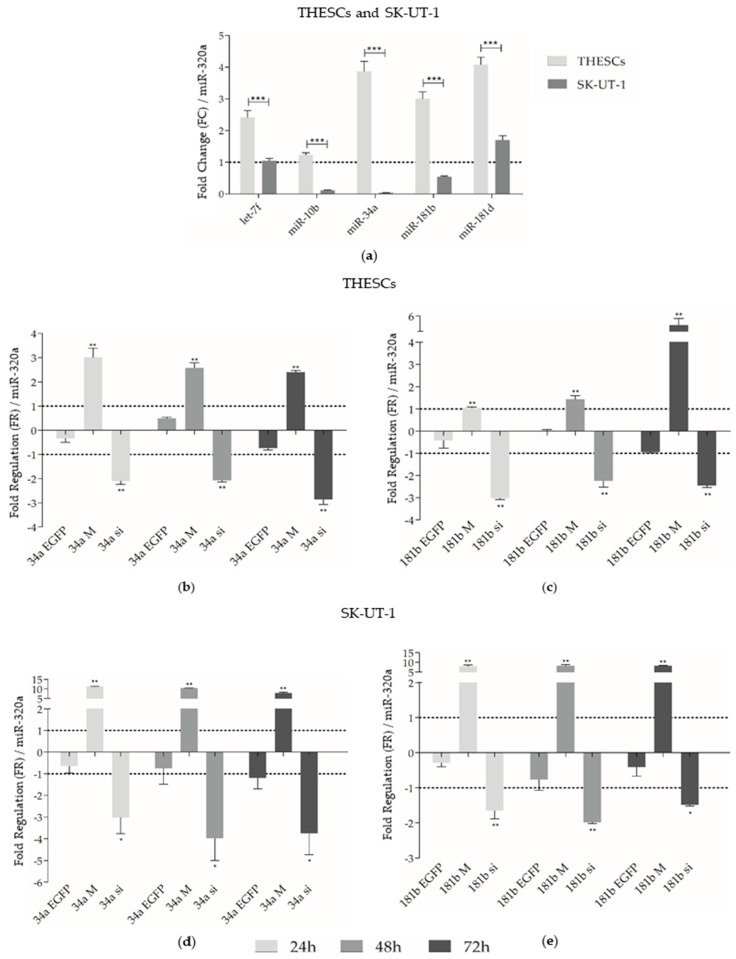
In vitro miRNA expression in both THESCs and SK-UT-1 cell lines. (**a**) Baseline transcript expression comparisons of *let-7f*, *miR-10b*, *miR-34a*, *miR-181b*, and *miR-181d* between THESCs and SK-UT-1 cells after normalization with *miR-320a* using PCS-460 cells as a reference. Statistically significant differences are represented by asterisks: *** *p* < 0.0001. (**b**) THESCs cells transfected with a *miR-34a* mimic and siRNA. *miR-34a* expression compared to EGFP siRNA 24, 48, and 72 h after transfection. (**c**) THESCs cells transfected with a *miR-181b* mimic and siRNA. *miR-181b* expression relative to EGFP siRNA 24, 48, and 72 h after transfection. (**d**) SK-UT-1 cells transfected with a *miR-34a* mimic and siRNA. *miR-34a* expression compared to EGFP siRNA 24, 48, and 72 h after transfection. (**e**) SK-UT-1 cells transfected with a *miR-181b* mimic and siRNA. *miR-181b* expression relative to EGFP siRNA 24, 48, and 72 h after transfection. Cells were analyzed by qPCR, using *miR-320a* as an endogenous control. Statistically significant differences between control and siRNA/mimic-transfected cells are represented by asterisks: * *p* < 0.05 and ** *p* < 0.005. 34a M: 34a mimic; 34a si: 34a siRNA; 34a EGFP, EGFP control for *miR-34a;* 181b M: 181b mimic; 181b si: 181b siRNA; 181b EGFP, EGFP control for *miR-181b*.

The miRNA expression profile was investigated in THESCs and SK-UT-1 cells, using the PCS-460 cells as a reference. The levels of *let-7f* (*p* < 0.0001), *miR-10b* (*p* < 0.0001), *miR-34a* (*p* < 0.0001), *miR-181b* (*p* < 0.0001), and *miR-181d* (*p* < 0.0001) expression were significantly lower in the SK-UT-1 cells compared to THESCs, as shown in Figure 1a.

Considering the molecular characteristics and clinical relevance of the miRNAs we reported previously [8,10,35,36], *miR-34a* and *miR-181b* were selected for in vitro functional validation in the present work. Although upregulation was observed in LM and downregulation in LMS, we decided to perform induction (mimics) and inhibition (siRNA) in both tumors. Therefore, THESCs and SK-UT-1 cells were transfected with *miR-34a* and *miR-181b* mimics and siRNA. The efficiency of transfected THESCs and SK-UT-1 cells was assessed using qPCR (Figure 1b–e). THESCs cells transfected with the 34a mimic showed enhanced expression of *miR-34a* (FR: 3.00) after 24 h, which was still detectable 72 h after transfection (Figure 1b). In these same cells, the 181b mimic induced higher expression levels of *miR-181b* (FR: 4.82) after 72 h (Figure 1c).

Efficient silencing of *miR-34a* and *miR-181b* were detected 24 h after transfection in the THESCs. The 181b siRNA inhibited *miR-181b* expression in 88% of cells after 24 h of transfection (FR: −3.02) and was still detectable at 72 h post-manipulation. The same expression profile was observed for 34a siRNA, with a lower expression level of *miR-34a* (FR: −2.86) detected 72 h (Figure 1b) after cell manipulation (86% efficiency of inhibition).

In SK-UT-1 cells transfected with 34a and 181b mimics (Figure 1d,e), the expression levels of *miR-34a* and *miR-181b* had a huge enhancement in expression at 24 (FR: 11.29 and FR: 8, respectively), 48 (FR: 10.43 and FR: 8.36), and 72 h (FR: 7.88 and 8.32). The silencing of *miR-34a* in these cells was detectable after 24h and led to lower expression of this miRNA 48 h after manipulation (FR: −3.98, efficiency of 84%) (Figure 1d). The SK-UT-1 cells transfected with 181b siRNA showed lower expression levels of *miR-181b* after 48h (FR: −1.98), with an efficiency of 75% (Figure 1e).

**Figure 2:**
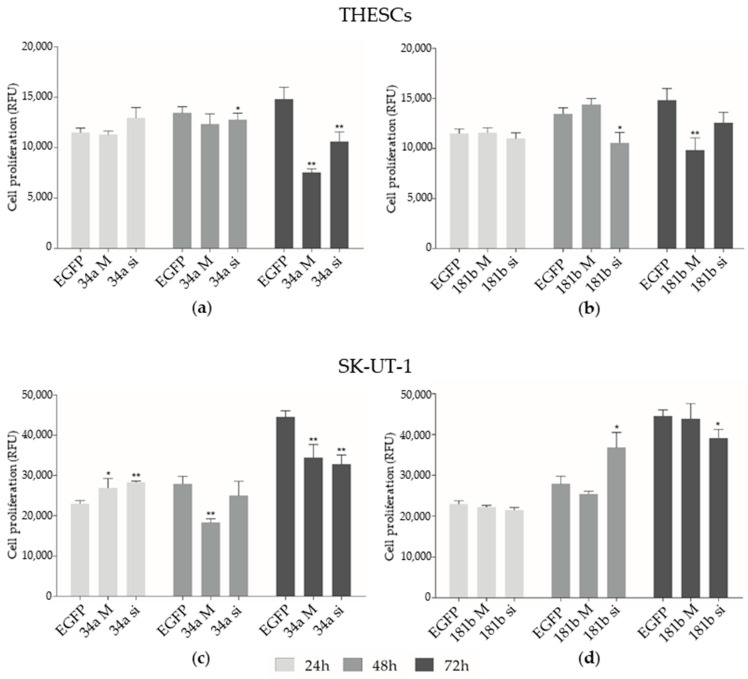
Proliferation assessment in THESCs (LM) and SK-UT-1 (LMS) cell lines. (**a**) Cell proliferation rate after transfections with 34a M (mimic) and 34a si (siRNA) showed significant differences compared to EGFP siRNA at 48 and 72 h in the THESCs cells. (**b**) Cell proliferation rate after transfections with 181b M (mimic) and 181b si (siRNA) showed significant differences compared to EGFP siRNA at 48 and 72 h in the THESCs cells. (**c**) Cell proliferation rate after transfections with 34a M (mimic) and 34a si (siRNA), with significant differences compared to EGFP siRNA at 24, 48, and 72 h, in SK-UT-1 cells. (**d**) Cell proliferation rate after transfections with 181b M (mimic) and 181b si (siRNA), with significant differences compared to EGFP siRNA at 48 and 72 h, in SK-UT-1 cells. Statistically significant differences between EGFP siRNA and transfected cells are represented by asterisks: * *p* < 0.05 and ** *p* < 0.005. RFU: relative fluorescence units. 34a M: 34a mimic; 34a si: 34a siRNA; 181b M: 181b mimic; 181b si: 181b siRNA.

Significant decreases in cell viability (proliferation assay) were observed in THESCs cells transfected with 34a siRNA after 48 h (*p* < 0.05) and up to 72 h (*p* < 0.005), diminishing cellular proliferation by 28% (Figure 2a). Transfection with 181b siRNA reduced cell proliferation by 22% at 48 h (*p* < 0.005); (Figure 2b). In these cell lines, transfection with 34a and 181b mimics inhibited proliferation in 49% and 34% of cells, respectively, after 72 h, compared to EGFP siRNA (*p* < 0.005).

In SK-UT-1 cells, transfection with the 34a mimic (*p* < 0.05) and siRNA (*p* < 0.005) increased cell proliferation in 23% of cells as early as 24h after transfection (Figure 2c). Cellular transfection with 181b siRNA significantly induced proliferation in 32% of cells after 48 h (Figure 2d). However, the 34a mimic led to a reduction in cell proliferation in 35% of cells after 48 h (*p* < 0.005) and up to 72 h (*p* < 0.005). In addition, transfection with 34a (*p* < 0.005) and 181b (*p* < 0.05) siRNA led to a significant inhibition of proliferation at 72 h, reducing it by approximately 23% compared to the EGFP siRNA control group (Figure 2c,d).

**Figure 3:**
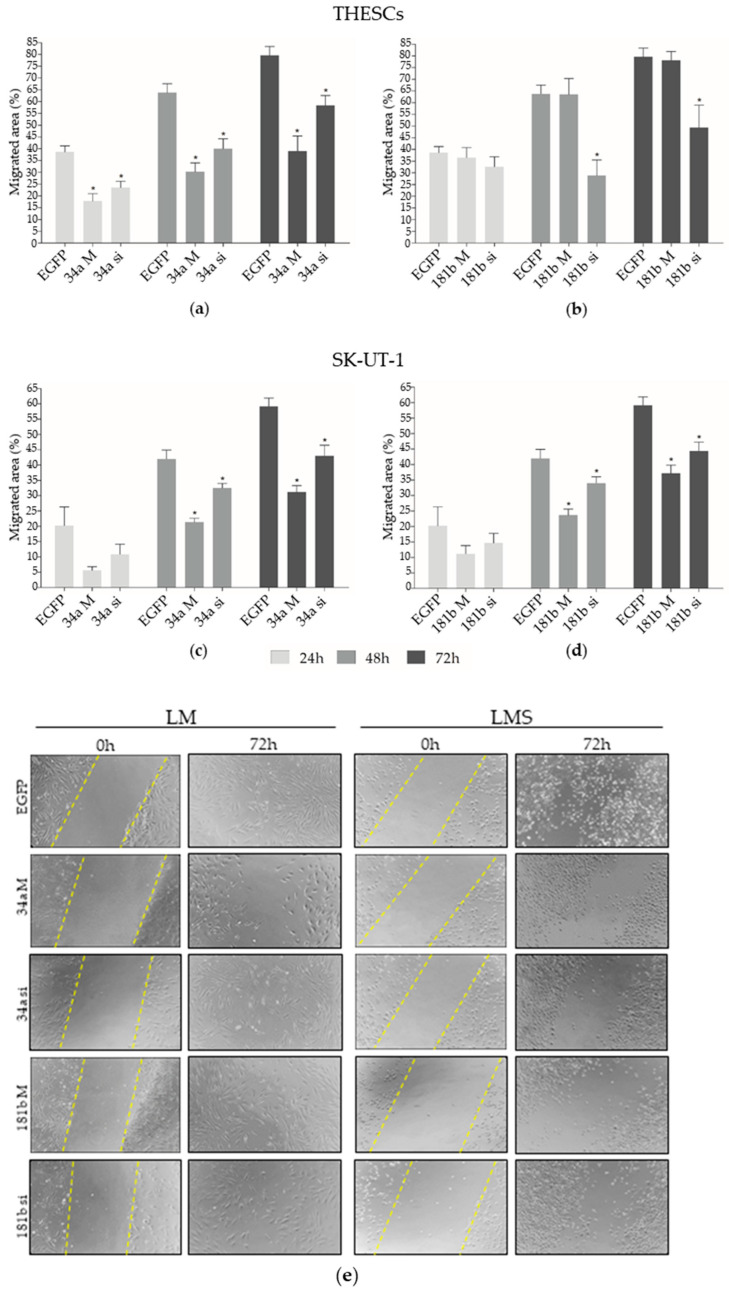
Migration assessment in THESCs (LM) and SK-UT-1 (LMS) cell lines. (**a**) Migration assay with THESCs cells transfected with 34a M (mimic) and 34a si (siRNA) with significant differences compared to EGFP siRNA at 24, 48, and 72 h. (**b**) Migration assay with THESCs cells transfected with 181b M (mimic) and 181b si (siRNA). The 181b si presented significant differences compared to EGFP siRNA at 48 and 72 h. (**c**) Migration assay with SK-UT-1 cells transfected with 34a M (mimic) and 181b si (siRNA). The cells showed significant differences for both 34a M and 34a si at 48 and 72 h, relative to EGFP siRNA. (**d**) Migration assay with SK-UT-1 cells transfected with 181b M (mimic) and 181b si (siRNA). The cells showed significant differences for both 181b M and 181b si at 48 and 72 h relative to EGFP siRNA. (**e**) Representative images (magnification of 10×) of THESCs and SK-UT-1 cells after scratching at 0 h and 72 h after transfections. The yellow dashed lines indicate the scratch region. Statistically significant differences between EGFP siRNA and transfected cells are represented by asterisks: * *p* < 0.05. 34a M: 34a mimic; 34a si: 34a siRNA; 181b M: 181b mimic; 181b si: 181b siRNA.

Cell migration was also assessed in transfected cells that exhibited a significant reduction in migration at 24 h and up to 72 h after 34a mimic transfection (*p* < 0.05). THESCs cells transfected with 34a siRNA had their migratory capacity impaired over 24, 48, and 72 h (*p* < 0.05), while the cells transfected with 181b siRNA showed a significant inhibition effect at 48 and 72 h compared to EGFP siRNA (*p* < 0.05; Figure 3a,b). Additionally, a significant reduction in SK-UT-1 cell migration was seen at 48 and 72 h after 181b mimic, 34a, and 181b siRNA transfections (*p* < 0.05), as compared to EGFP siRNA (Figure 3c,d). Cells transfected with EGFP siRNA exhibited complete scratch closure 96h after transfection.

**Figure 4:**
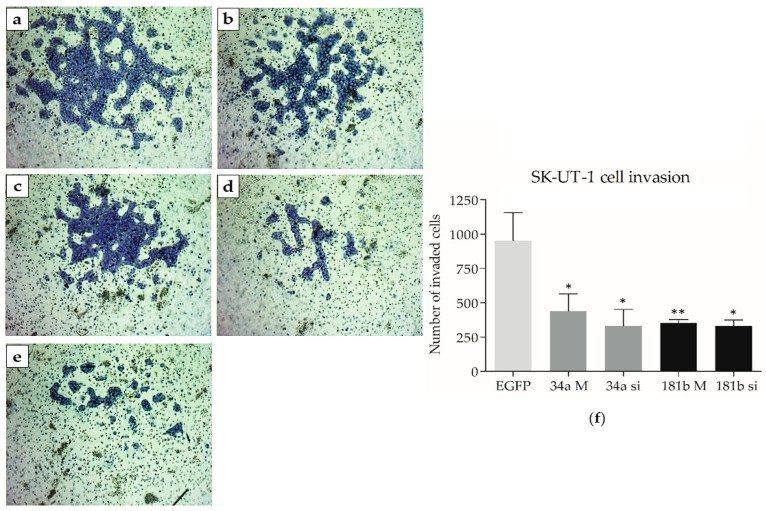
Invasion assay using SK-UT-1 cells after transfection with 34a and 181b mimics and siRNA (magnification of 4×). (**a**) EGFP siRNA-transfected cells. (**b**) Cell invasion with the *miR-34a* mimic. (**c**) Cell invasion with *miR-34a* siRNA. (**d**) Cell invasion with the *miR-181b* mimic. (**e**) Cell invasion with *miR-181b* siRNA. (**f**) The graph shows significant differences in the number of invading cells between EGFP siRNA and cells transfected with 34a and 181b mimics and siRNA. Statistical differences between EGFP siRNA and transfected cells are represented by asterisks: * *p* < 0.05 and ** *p* < 0.005. 34a M: 34a mimic; 34a si: 34a siRNA; 181b M: 181b mimic; 181b si: 181b siRNA.

The functional role of *miR-34a* and *miR-181b* in the invasion process was investigated in SK-UT-1 LMS cells (Figure 4). A significant inhibitory effect on the invasion ability of SK-UT-1 cells was observed after 34a (*p* = 0.0041) and 181b (*p* = 0.0002) mimic and 34a (*p* = 0.0017) and 181b (*p* = 0.0008) siRNA transfections, as compared to EGFP. On the other hand, transfection with *miR-34a* and *miR-181b* siRNAs/mimics did not induce the invasion ability of THESCs cells.

**Figure 5:**
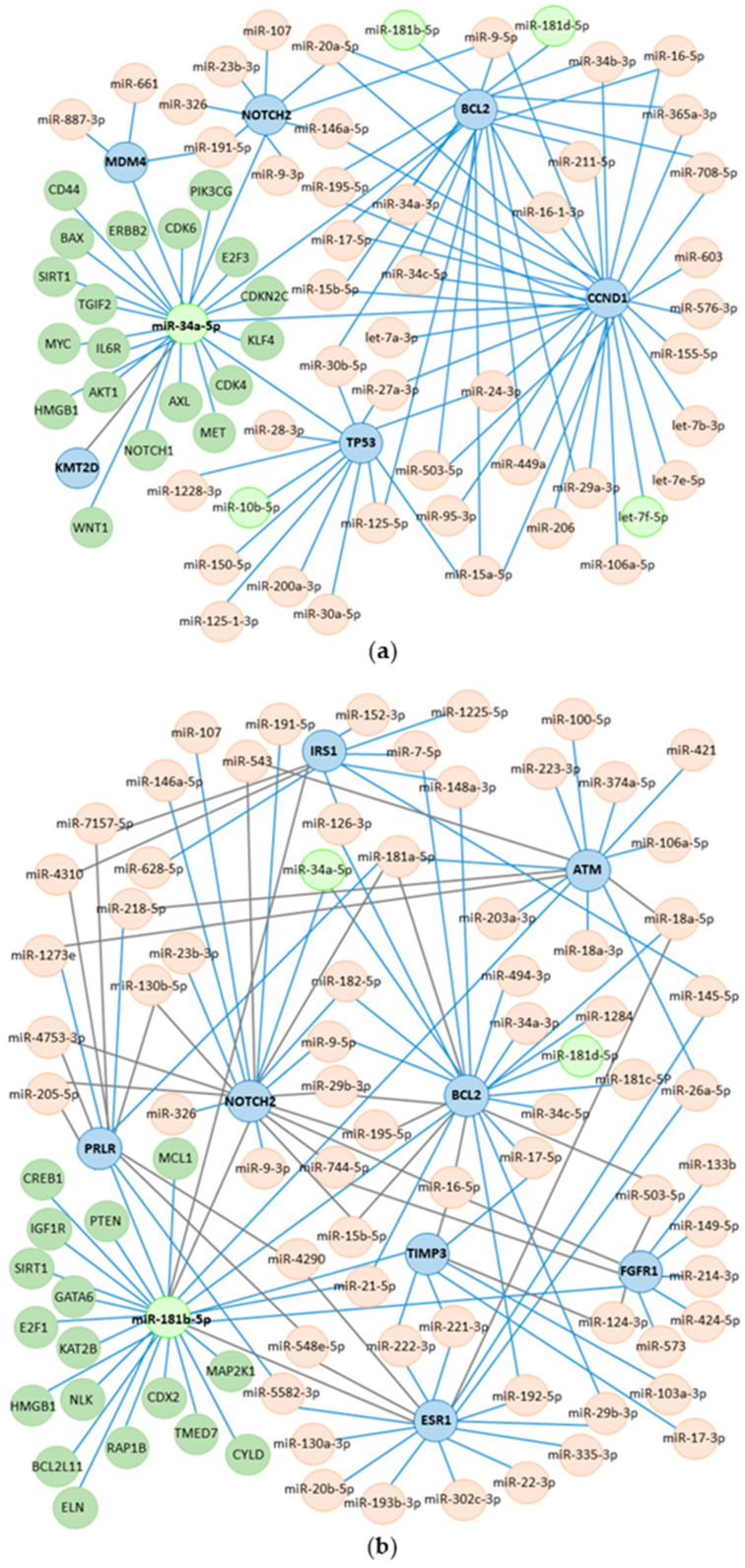
The miRNA–mRNA and miRNA–miRNA interactions of *miR-34a* and *miR-181b* [43,44,45,46,47]. (**a**) The network of *miR-34a-5p* with target genes evaluated in the study, *MDM4*, *NOTCH2*, *BCL2*, *CCND1*, *TP53* and *KMT2D*. (**b**) The network of *miR-181b-5p* with target genes evaluated in the study, *TIMP3*, *FGFR1*, *ATM*, *IRS1*, *PRLR*, *ESR1*, *BCL2*, and *NOTCH2.* The light green color denotes the miRNAs selected for this study. Shown in green, some important target genes are regulated by miRNA. The target genes selected for this study are indicated in blue, and the light pink color shows other miRNA interactions. Blue lines indicate targets with strong validation, and grey lines indicate weak validation targets.

**Figure 6:**
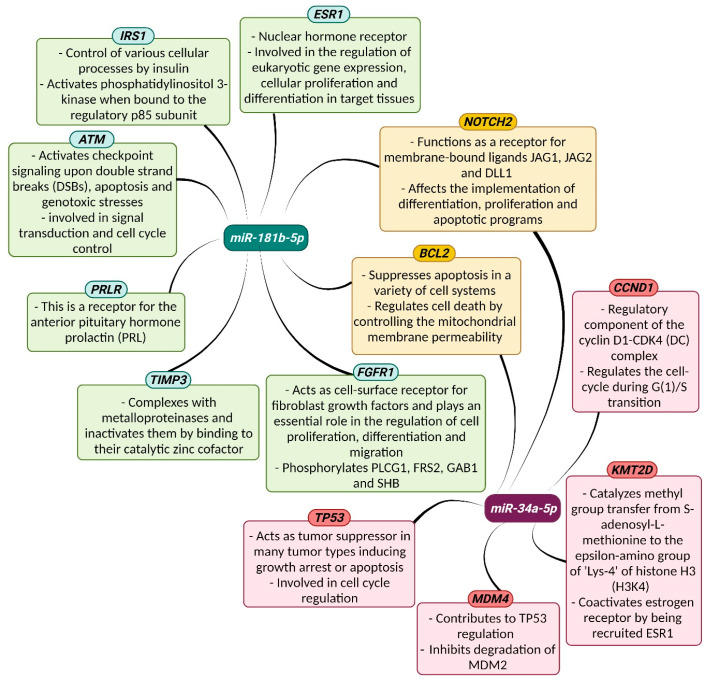
Genetic interactions of *miR-34a* and *miR-181b* and predicted targets [48]. *MiR-34a* is a potential modulator of *CCND1*, *KMT2D*, *MDM4*, *TP53*, *BCL2*, and *NOTCH2* expression (purple). However, *BCL2* and *NOTCH2* are also regulated by *miR-181b* (beige). *MiR-181b* is a potential modulator of *TIMP3*, *FGFR1*, *ATM*, *IRS1*, *PRLR*, and *ESR1* expression (green).

To assess the effects of the transfections at the post-transcriptional level, an in silico analysis was performed to identify the predicted target genes of *miR-34a* and *miR-181b* (Figure 5). The *miR-34a* network showed the potential regulation of genes involved in cell cycle control, apoptosis, senescence, DNA repair, transcriptional regulation, membrane receptors, and methylation. Genes potentially regulated by *miR-181b* include insulin receptors, hormonal receptors, cell cycle control, apoptosis, proliferation, differentiation, mitosis, and extracellular matrix degradation. We selected key *miR-34a*-targeted genes (*CCND1*, *KMT2D*, *MDM4*, *TP53*, *BCL2*, and *NOTCH2)* and *miR-181b*-targeted genes *(TIMP3*, *FGFR1*, *ATM*, *IRS1*, *PRLR*, *ESR1*, *BCL2*, and *NOTCH2)*, shown in Figure 6, for further analysis.

RNA expression levels of target genes were assessed using qPCR analysis at 48 and 72 h after transfection in THESCs and SK-UT-1 cells, as shown in Table 1. Genes including *MDM4*, *TP53*, and *CCND1* presented significant increases in expression after transfection with the 34a mimic, as compared to EGFP siRNA, in THESCs and SK-UT-1 cells. However, a decrease in *KMT2D* and *NOTCH2* expression was observed in both cell lines after transfection with the 34a mimic.

In the SK-UT-1 cells, the expression levels of *CCND1*, *KMT2D*, *MDM4*, *TP53*, *BCL2*, and *NOTCH2* were upregulated after 34a siRNA transfection, while *BCL2* and *MDM4* were significantly increased in THESCs relative to EGFP siRNA. *KMT2D* and *NOTCH2* exhibited significant reductions after the silencing of *miR-34a* in THESCs.

*NOTCH2*, *ATM*, and *IRS1*, as target genes of *miR-181b*, showed significantly reduced expression levels in THESCs and SK-UT-1 cells after 181b mimic and siRNA transfections, as compared to EGFP siRNA.

Transfection of THESCs cells with the 181b mimic led to a significant increase in *PRLR* and *FGFR1* expression and decreased *BCL2* expression. In SK-UT-1 cells, 181b mimic transfection resulted in increased *FGFR1* expression and reduced expression of *TIMP3*, respectively.

In addition, transfection of THESC cells with 181b siRNA increased the expression of *TIMP3* and *FGFR1* and decreased the expression of *PRLR* compared to EGFP. *BCL2* presented a significant decrease in THESCs and SK-UT-1 cells after silencing of *miR-181b*, as compared to EGFP. SK-UT-1 cells did not express *PRLR* and *ERS1* even after mimic transfections (Table 1).

**Figure 7:**
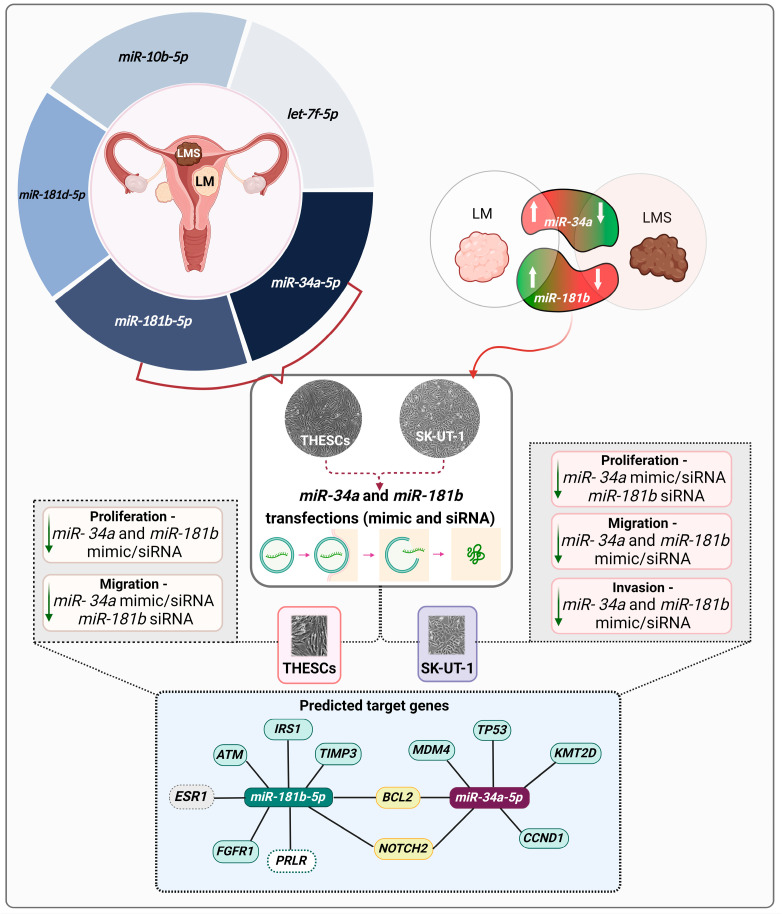
Representative diagram of the present study’s main results. Our group previously investigated the five miRNAs selected for this study in patient samples. The *miR-34a-5p* and *miR-181b-5p* were selected for functional validation in THESCs (LM, benign tumor) and SK-UT-1 (LMS, malignant tumor) cells, which were transfected with mimic and siRNA. The effects of molecular manipulation reduced cell proliferation, migration, and invasion. After transfection, the expression profile of a set of target genes was assessed. Genes potentially regulated by *miR-34a-5p* and *miR-181b-5p* are presented in light blue. Genes potentially regulated by both miRNAs are in yellow. Genes potentially regulated by *miR-181b-5p* only in THESCs cells are in white. Genes not regulated by *miR-181b-5p* in both cells are in grey.

Based on the obtained data, aberrantly low expression of *miR-34a* and *miR-181b* might contribute to the development of LMS and poor prognosis in patients, as schematically represented in Figure 7. Our in vitro findings in this study suggest that the loss of function of *miR-34a* and *miR-181b* is associated with an increase in cell survival, migration, and invasion, which may contribute to the progression of LMS.

## 4. Discussion

All the miRNAs in this study were selected due to their specific target genes, which are involved in tumor pathogenesis or key cellular processes [28,49,50,51]. In this study, we first observed that *let-7f*, *miR-10b*, *miR-34a*, *miR-181b*, and *miR-181d* had lower expression levels in SK-UT-1 cells compared to THESCs. These results suggested that the five miRNAs might be targets of extensive studies as potential biomarkers in the characterization of the differentiation of benign and malignant UMTs, in which the loss or inactivation of these miRNAs may contribute to malignant tumor progression [52].

In a previous screening study [35], *miR-181b* exhibited lower expression levels in endometrial stromal sarcoma, carcinosarcoma, and LMS. Downregulation of *let-7f*, *miR-10b*, and *miR-34a* was associated with worse prognoses and aggressiveness in gynecological cancers [10,35,36,49,50,53,54,55,56,57]. Based on preliminary results, we selected *miR-34a* and *miR-181b* for the functional validation assays, focusing on their clinical relevance as described in several other tumors. Although we observed that *miR-34a* and *miR-181b* were upregulated in LM and downregulated in LMS cells, we decided to perform transfections with mimics and siRNA in both cells. However, our main goal was to elucidate the effects of loss- and gain-of-function approaches, purely for biological effect analyses of basal expression reversion of these miRNAs.

Gain of function of *miR-34a* resulted in proliferation, migration, and invasion capability inhibition in SK-UT-1 cells. In cervical and breast cancer cells, the introduction of miR-34a had similar effects [58,59]. Additionally, *miR-34a*-induced apoptosis was observed in several types of cancer [58,60], corroborating our findings. Conversely, *miR-34a* silencing in malignant tumors can promote cell invasion and migration, as described by Bao et al. in colorectal cancer [61]. Although *miR-34a* siRNA suppressed proliferation and migration in THESCs, we did not observe that *miR-34a* was able to promote LM cell invasion.

*MiR-181b*’s role in inflammatory processes and malignant transformation has aroused interest [62]. We saw that the suppression of *miR-181b* suppressed the ability of SK-UT-1 cells to migrate and invade, whereas its high expression promotes proliferation and migration in vascular smooth muscle cells and breast cancer [63,64,65]. In addition, the *miR-181b* mimic increased hypertrophic scar fibroblast proliferation and reduced apoptosis [66]. The precise function of this miRNA appears to be context-dependent, varying according to the tumor type and malignancy and with conflicting roles [67,68].

Studies focusing on potential specific targets have shown *miR-34a* and *miR-181b* interactions with a wide range of oncogenes [22,27,28,69,70,71,72,73], which might reveal a regulatory cycle of miRNA–gene/gene–miRNA interactions [27,28,74]. Regulatory mechanisms work differently depending on the cell type and whether conditions are disease-related. Additionally, how mature miRNAs affect gene activity depends on various factors, such as the subcellular location of miRNAs, the affinity of miRNA–mRNA interactions, and the abundance of miRNAs and target mRNAs. As we showed in the in silico analysis, these molecules have hundreds of mRNA targets, and, in contrast, one single mRNA can be regulated by many miRNAs [74,75].

Effects of *miR-34a* on tumor suppression are closely associated with the regulation of *TP53*, *Wnt/β-Catenin*, *JAK2/STAT3*, *PI3K/AKT/BIRC5*, and *NOTCH* signaling pathways [26,76]. *MiR-181b* was also associated with some of these pathways’ regulation [28,73,77,78,79]. Key genes of *NOTCH*, *TP53*, *PRL*, *ER*, *INS*, and apoptosis pathways, such as *KMT2D*, *FGFR1*, *TIMP3*, *MDM4*, *BCL2*, and *CCND1* [80,81,82,83], were assessed in the cell lines after their genetic manipulation.

In our study, *CCND1*, *KMT2D*, and *TP53* displayed increased expression levels after *miR-34a* delivery in SK-UT-1 cells. However, only *CCND1* and *MDM4* presented higher expression levels in THESCs cells after *miR-34a* silencing. Gain of function of *miR-34a* promotes the downstream effects of *TP53*. In part, these effects are mediated by the post-transcriptional repression of targets such as *CCND1*, *CCNE2*, *CDK4*, *MET*, *MYC*, *BCL2*, *SNAIL1*, and *SIRT1*, which stimulate multiple oncogenic processes [27,84]. *MiR-34a* represses *MDM4* [85], *CCND1* [72], and *BCL2*, which are directly or indirectly involved in the *TP53* pathway [86]. Although *TP53* is a *miR-34a* target, a feed-forward loop was described, showing that *TP53* induces *miR-34* family transcription and that *miR-34a* suppresses multiple *TP53* inhibitors [87]. In these positive and negative feedback mechanisms, *TP53* activity produces a robust tumor suppression response [27,84,87]. Ectopic restoration of *miR-34a* reduced *BCL2* and *SIRT1* expression, indicating an inhibitory effect of this miRNA on *TP53* [58,88]. The decrease in *SIRT1* mediated by *miR-34a* seems to induce cell cycle arrest and sensitivity to chemotherapy. Additionally, an inverse correlation between *SIRT1* and *miR-34a* expression was observed in prostate cancer, which led to drug resistance through the *BCL2* pathway [89]. In our cell lines, the *miR-34a* mimic reduced *BCL2* and *NOTCH2* levels. Córdova-Rivas et al. found that these miRNAs induced apoptosis through *BCL2* inhibition in cervical cancer cells [59]. In addition, *miR-34a* has an inhibitory effect on *NOTCH1*, *NOTCH2*, and *NOTCH4* expression in pancreatic ductal carcinoma [90], whereas, in colon carcinoma, its inhibition rescued the expression of *NOTCH1*, *NOTCH2*, and *BCL2* [91].

Although previous bioinformatic analysis indicated the interaction of *KMT2D* with *miR-34a* [71], experimental validation has not been conducted yet. In this study, we revealed a positive correlation between *miR-34a* and *KMT2D. miR-34a* silencing decreased *KMT2D* expression in THESCs cells, while the delivery of *miR-34a* led to an increased expression of *KMT2D* in SK-UT-1 cells.

Hence, the action and participation of *miR-34a* are relevant for several molecular processes, including anti-tumor actions. An in-depth understanding of the upstream and downstream pathways of miRNA would be a prerequisite for its successful therapeutic application. Regulatory cycles may be substantially affected by the therapeutic dose of the *miR-34a* mimic. Thus, *miR-34a* can lead to potential anti-cancer effects on therapy based on this molecule, mainly when *TP53* presents an interrupted or impaired function [27].

The roles of *miR-181b* may be unique depending on the tumor type and cellular context. This miRNA regulates genes involved in cell signaling, cycle control, and chemosensitivity in cells harboring *TP53* mutations or deletions [28]. It can act as a critical component in inflammation, tumorigenesis, chemosensitivity, and apoptosis by suppressing the expression of *TIMP*, *TP53*, and *BCL2*. *miR-181b* is related to aging, peritoneal cavity homeostasis, and insulin resistance [62,92,93,94].

Here, we found that the introduction of *miR-181b* decreased the expression of *NOTCH2*, *TIMP3*, *ATM*, and *IRS1* while increasing the expression of *FGFR1* in SK-UT-1 cells. On the other hand, *miR-181b* silencing promoted *TIMP3* and *FGFR1* expression in THESCs cells, while the expression of *NOTCH2*, *ATM*, *IRS1*, *PRLR*, and *BCL2* was inhibited. Previous studies reported that the delivery of *miR-181b* led to an inhibitory effect on the expression of several tumor suppressor genes, including *CYLD*, *LATS2*, *NDRG2*, and *TIMP3*. This miRNA may contribute to tumor growth, metastasis, and EMT induction in several cancer types by suppressing *TIMP3* [88,95,96,97,98].

*MiR-181b* exhibits a dual role by counteracting the oncogenic effects of *NOTCH2* [99]. In our investigation, *miR-181b* manipulation, either through introduction or silencing, decreased *NOTCH2* expression in both cell lines. This modulation effectively curbed the *NOTCH2*-induced enhancement of cell invasiveness and proliferation [99,100]. The clinical relevance of *miR-181b* and *NOTCH2* was observed in cell lung cancer, where their co-regulation influences patient prognosis and survival outcomes [99]. *NOTCH2* has a dual role, acting as an oncogene and tumor suppressor in different contexts [70,101]. Concomitantly, *miR-181b* manipulation yields a remarkable outcome, leading to a discernible decrease in *ATM* expression in THESCs and SK-UT-1 cells. *ATM* is a key regulator of essential cell cycle control and DNA damage response [102,103]. This finding aligns with prior research, which linked *miR-181b* to aggressive breast tumors due to its capacity to downregulate *ATM* [103]. In addition, TGF-β treatment implicates *ATM* suppression and induction of *miR-181b* expression, emphasizing the complexity of *ATM* regulation [104].

In our study, *PRLR* reduction was observed in 181b-siRNA-transfected THESCs cells. *PRLR* has been demonstrated to play an oncogenic role in prostate, breast, cervical, ovarian, and pancreatic tumors. The greater expression of *PRLR* drives proliferation, migration, and invasion processes [105]. *PRL* binding to its receptor induces downstream signaling that includes *JAK-STAT*, *PI3K/AKT*, *RAF/MEK/ERK*, and *MAPK* pathways [106]. The interconnected circuits and crosstalk of these pathways may promote tumor development, progression, and metastasis [107]. Therapeutic strategies attempt to modulate *PRLR* activity by suppressing downstream signaling or through antagonists [105,108]. Considering the action mechanisms of *miR-181b* and the *PRLR* regulatory cycle, a compensatory effect may occur on the treatment response. *PRLR* regulation by *miR-181b* in cancers needs to be elucidated since both play important roles in cellular metabolism.

Pro-angiogenic *FGF1* and *uPA* genes were suppressed by *miR-181b* in the extracellular vesicles of human liver stem cells, indicating its anti-angiogenic action [109]. While the exact mechanism of the direct regulation of *FGFR1* by *miR-181b* is not fully understood, it is well-established that the *FGF/FGFR* interaction triggers FGFR dimerization and activation, subsequently activating *AKT/PI3K*, *MAPKs*, *PLCγ/PKC*, and *STATs* pathways. These pathways control differentiation, migration, apoptosis, and the cell cycle, and their dysregulation often promotes cancer and drug resistance [110]. We observed higher levels of *FGFR1* expression following *miR-181b* delivery in malignant tumor cells. Conversely, 181b siRNA also increased *FGFR1* expression in THESCs cells. *FGFR1* overexpression can drive tumorigenesis by enhancing cell proliferation and migration and inhibiting cell death. Combining an *FGFR1* blockade with other anti-cancer drugs can be an interesting strategy for cancers that exhibit abnormal *FGFR1* expression [111].

Although LMS is a rare neoplasia, the precise diagnosis of LMS and LM is possible only after surgery [112]. Specific biomarker identification might allow the differential diagnosis of benign and malignant tumors before surgeries. However, further studies could be necessary to determine the role of *miR-34a* and *miR-181b* in UMT differentiation. Most research carried out to date has not identified both simultaneously. More specifically, the expression differences of miRNAs between LM and LMS have not been elucidated [5], restricting the understanding of action mechanisms implicated in pathogenesis and progression.

Therapies focusing on recovering tumor suppressor proteins and inhibiting oncogenic ones by regulating miRNAs need to be further explored, which can effectively modulate pathological processes, avoiding undesirable toxic effects in normal tissues [8,113]. Epigenetic treatment can bring beneficial effects through monotherapy or combination treatment with other standard therapies [22]. Nevertheless, the therapy’s success may depend on factors such as delivery systems and miRNA degradation. Additionally, potential side effects and immune responses cannot be ruled out [26]. Finally, knowledge of the mutual regulation of miRNA/target genes contributing to uterine disorders still needs to be improved. miRNA-specific effects evaluated in UMTs might be the focus of studies in developing therapies and diagnostic tools as potential biomarkers.

## 5. Conclusions

In this study, we characterize the role of *miR-34a* and *miR-181b* in LM and LMS cells. Our functional studies revealed that these two miRNAs are crucial in impacting cell proliferation and migration in two different types of cells. Notably, a single introduction of either a *miR-34a* or *miR-181b* mimic resulted in an inhibitory effect on LMS cell invasion ability. Concomitantly, *miR-34a/miR-181b*-targeted genes have been identified, shedding light on the molecular mechanism underlying miRNA-related uterine tumor pathogenesis. Investigating these miRNAs and their target genes offers a promising approach to LM and LMS differentiation. Incorporating miRNA modulation in tumor therapy or even applying it to prognoses could provide new perspectives on the outcomes of LMS patients. Further studies are needed to better understand the mechanisms underlying the anti-oncogenic roles of *miR-34a* and *miR-181b* in LMS.

## Figures and Tables

**Table 1:** Comparisons of transcript expression levels of target genes regulated by *miR-34a* and *miR-181b*.

miRNA (mimic/siRNA)		Target Genes	THESCs		SK-UT-1	
			48 h (FC)	72 h (FC)	48 h (FC)	72 h (FC)
*miR-34a*	34a mimic	*CCND1*	0.91 *	0.86 *	1.85 **	1.23 **
		*KMT2D*	0.75 **	0.73 **	1.20	0.97 **
		*MDM4*	1.33 *	1.31 *	1.29	0.98
		*TP53*	0.92 **	0.78	1.05 *	0.78 *
		*BCL2*	0.43 **	0.62 **	1.27 *	1.63
		*NOTCH2*	0.42 **	0.41 **	0.78 **	0.59
	34a siRNA	*CCND1*	1.28	1.05 *	12.84 **	1.20 **
		*KMT2D*	0.47	0.52 **	1.23 *	0.87 **
		*MDM4*	0.80	1.26 *	5.81 **	1.29
		*TP53*	0.69	0.73	4.33 **	0.95
		*BCL2*	0.86 **	0.79 *	7.13 **	1.85
		*NOTCH2*	0.47	0.45 **	1.11 **	0.68
*miR-181b*	181b mimic	*TIMP3*	0.57	0.65	0.72 *	0.92
		*FGFR1*	0.77	0.97 *	1.06	1.19 **
		*ATM*	0.34 *	0.45 **	0.64 **	0.41 *
		*IRS1*	0.54 *	0.61 **	0.67 *	0.63
		*BCL2*	0.56 *	0.42 **	2.27	2.28
		*NOTCH2*	0.33 **	0.34 **	0.72 **	0.59 **
		*ESR1*	0.99	0.81	-	-
		*PRLR*	0.94 *	1.14 *	-	-
	181b siRNA	*TIMP3*	0.53	0.91 *	0.82	0.92
		*FGFR1*	0.75	0.87 *	1.07	0.93
		*ATM*	0.27 **	0.35 **	0.70 *	0.43 *
		*IRS1*	0.37 **	0.50 **	0.61 **	0.43 **
		*BCL2*	0.38	0.33 **	1.67 **	1.44
		*NOTCH2*	0.23 **	0.27 **	0.78 **	0.49 **
		*ESR1*	0.67	1.14	-	-
		*PRLR*	0.63	0.75 *	-	-

Downregulated genes after transfections are shown in blue and upregulated genes in red, relative to EGFP siRNA. The colors indicate an increase or decrease in gene expression levels compared to EGFP expression and do not refer to the cut-off value. Statistically significant differences between EGFP siRNA and transfected cells are represented by asterisks: * *p* < 0.05 and ** *p* < 0.005. FC, fold change.

## Data Availability

All data generated or analyzed during this study are included in this article or the Supplementary Materials.

## Supplementary Materials

The following supporting information can be downloaded at: https://www.mdpi.com/article/10.3390/biomedicines13030560/s1, Table S1: TaqMan inventoried probes for miRNAs’ target genes detection by qPCR.

## Abbreviations

The following abbreviations are used in this manuscript:

LM	Leiomyoma
LMS	Leiomyosarcoma
UMT	Uterine mesenchymal tumors
FC	Fold change
FR	Fold regulation
