# Supplementary material for: Could let-7f, miR-10b, miR-34a, miR-181b, and miR-181d Be Useful Tools as a Target Therapy for Uterine Leiomyosarcoma?
Source: Biomedicines. 2025 Feb 23;13(3):560. doi: 10.3390/biomedicines13030560 (PMC11940384; doi:10.3390/biomedicines13030560)
Supplement: Supplementary file 1 [file biomedicines-13-00560-s001.zip › biomedicines-3458693-supplementary.pdf]

Table S1. TaqMan inventoried probes for miRNAs' target genes detection by qPCR.

miRNA	Target gene	Reference
miR-34a-5p	CCND1	HS00765553_m1
	BCL2	HS00608023_m1
	KMT2D	HS00912416_m1
	MDM4	HS00159092_m1
	TP53	HS01034249_m1
	NOTCH2	HS01050702_m1
miR-181b-5p	TIMP3	HS00165949_m1
	FGFR1	HS00241111_m1
	ESR1	HS01046816_m1
	BCL2	HS00608023_m1
	NOTCH2	HS01050702_m1
	ATM	HS00175892_m1
	PRLR	HS01061477_m1
	IRS1	HS00178563_m1
